# An Anti-Jamming Null-Steering Control Technique Based on Double Projection in Dynamic Scenes for GNSS Receivers

**DOI:** 10.3390/s19071661

**Published:** 2019-04-07

**Authors:** Hao Wang, Qing Chang, Yong Xu

**Affiliations:** 1 School of Electronic and Information Engineering, Beihang University, No. 37 Xueyuan Road, Haidian District, Beijing 100191, China; buaahao@126.com; 2 Institute of Unmanned Systems Research, Beihang University, No. 37 Xueyuan Road, Haidian District, Beijing 100191, China; xuyong1518@163.com

**Keywords:** interference, GNSS, spatial filtering, projection, null steering

## Abstract

When a global navigation satellite system (GNSS) receiver suppresses interference in a dynamic scene, the direction of the interference signal arriving at the receiver may change rapidly. The null formed by the spatial filtering based on the array antenna will become wider and shallower, and the anti-jamming performance will deteriorate. A null-steering control technique based on a dual projection algorithm is proposed in this paper, which can effectively increase the depth of the null. In this paper, the dynamic model between the interference and the receiver is established first. Based on the model, the rate of change of the arrival direction of the interference is analyzed, and the phenomenon of the spatial filtering null becoming wider and shallower is simulated and verified. Then, the double projection algorithm is introduced to effectively deepen the null. The simulation results show that the proposed method can effectively increase the null depth by 30 to 50 dB, which significantly improves the anti-jamming performance of the spatial filtering in dynamic scenes.

## 1. Introduction

In complex electromagnetic environments or in dealing with battlefield electronic countermeasures, a satellite navigation receiver is confronted with interference intrusions [1,2]. The spatial domain filtering technique based on the array antenna can form nulls in the direction of interference to suppress interference, which is a mainstream anti-jamming technique [3,4,5,6]. In practice, there is often a relative motion between the interference and the receiver. The incident direction of the interference signal arriving at the receiver is constantly changing. When the relative motion causes the interference direction to change rapidly, such as in a missile flight scene or when there is a dynamic interference source, the null formed by the spatial filtering algorithm will not be able to eliminate the interference normally. This is because the arrival angle of the interference signal changes rapidly, and the signal sampled each time is also a dynamically changing signal; therefore, calculating the anti-jamming vector based on the sample covariance matrix derived from the sampled signal also forms a widened null. The depth of the null will also decrease, and the interference cannot be effectively eliminated, resulting in a sharp deterioration of the anti-interference performance. A method of interference-null control based on double projection is proposed in this paper, which can significantly increase the depth of the null, and ensure the anti-jamming performance of the spatial filtering in dynamic scenes. Regarding the null control technique, a curved array split into two subarrays was proposed in Ref. [7] to improve the pattern null depth and width for controlled reception pattern antenna systems. A method of projecting the autocorrelation matrix into the interference space was proposed in Ref. [8] to improve the anti-interference performance in the presence of coherent interference. In Ref. [9], a broadened model based on maximizing output power and constraining interference was proposed to improve the signal-to-interference and -noise ratio of the receiver. In Ref. [10], a novel statistical space–time null widening method was proposed to suppress interference in dynamic conditions, and Refs. [11,12] proposed the implementation of an adaptive null-steering beamformer for flexible broad null control. However, the simulation of dynamic scenes is lacking in these papers. In the actual dynamic scene, the generated null will naturally widen. The problem we face is actually how to deepen the null.

In this paper, we first establish a dynamic model and analyze which parameters are related to the rate of change of the interference direction. Then, the phenomenon that the null of the spatial filtering becomes wider and shallower in dynamic scenes is verified. Finally, the null control algorithm based on a double projection is introduced to improve the anti-interference performance of the spatial filtering in dynamic scenes by deepening the depth of the null. The simulation results show that the proposed method has better ability to control for interference nulls. The first projection can increase the depth of the null by at least 20–30 dB. After double projection, it can continue to increase by more than 20 dB.

The outline for the remainder of this paper is as follows. Section 2 presents the dynamic model and signal model. The analysis of the spatial filtering performance in static and dynamic scenes is presented in Section 3, and the null control algorithm based on double projection is presented in Section 4. Simulation results and conclusions are presented in Section 5 and Section 6, respectively.

## 2. Dynamic Model and Signal Model

**Figure 1:**
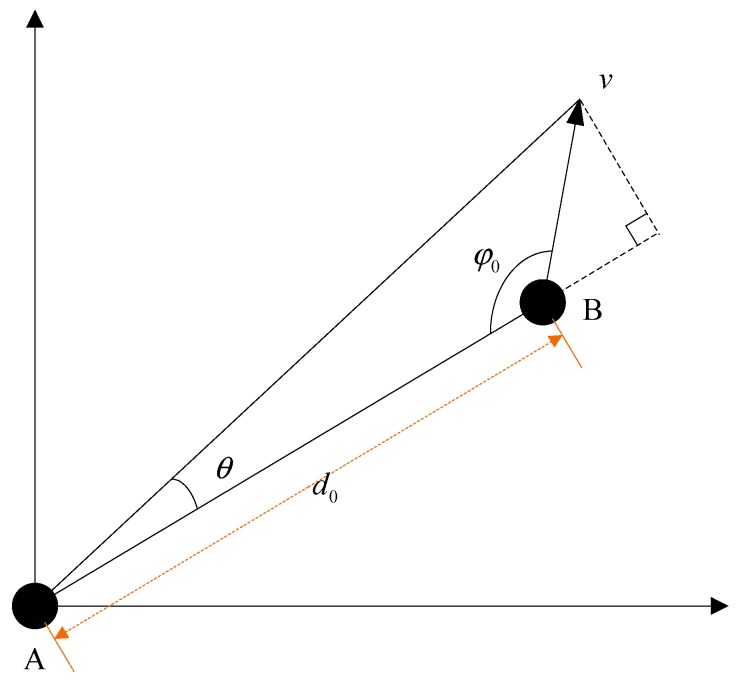
Schematic diagram of interference and receiver dynamic motion model.

As shown in Figure 1, we assume that A is the receiver carrier and B is the interference source. The initial distance between the receiver and the interference source is $d_{0}$. The angle between the velocity direction of the interference source relative to the receiver and the radial connection between them is $\varphi_{0}$, and $\left\{0^{{}^{\circ}}\leq \varphi_{0}\leq 180^{{}^{\circ}}\right\}$. We calculate the change in the direction of the arrival of the interfering signal, i.e., the change in the angle θ in Figure 1:

(1) $$\theta (t)=\arctan\left[\frac{v\cdot t\cdot \sin(\pi -\varphi_{0})}{d_{0}+v\cdot t\cdot \cos(\pi -\varphi_{0})}\right]$$

where represents the relative motion velocity, and represents time, and we take the derivative of θ(t) with respect to t and get

(2) $$\theta^{\prime }(t)=\frac{d_{0}\cdot v\cdot \sin\varphi_{0}}{{\left(d_{0}-v\cdot t\cdot \cos\varphi_{0}\right)}^{2}+{\left(v\cdot t\cdot \sin\varphi_{0}\right)}^{2}}$$

When the initial parameters of the motion are determined, $d_{0}$ and v must be positive, and $0^{{}^{\circ}}<\varphi_{0}<180^{{}^{\circ}}$, so $\theta^{\prime }(t)>0$. Therefore, θ(t) will gradually increase with time t, however, it can be obtained from Equation (2) that as time t increases, $\theta^{\prime }(t)$ will gradually decrease, so the rate of change of θ(t) will become increasingly small. In special cases, when $\varphi_{0}=0^{{}^{\circ}}$ or $\varphi_{0}=180^{{}^{\circ}}$, $\theta^{\prime }(t)=0$, and at this time, θ(t)≡0.

Regarding the relationship between the rate of change of the interference direction and the relative motion speed v between the interference and the receiver, the initial distance $d_{0}$ and the relative motion direction $\varphi_{0}$, we separately take the partial derivatives of $\theta^{\prime }(t)$ with respect to v, $d_{0}$, and $\varphi_{0}$.

First, we take the partial derivative of $\theta^{\prime }(t)$ with respect to v, and it can be obtained from Equation (2),

(3) $$\frac{\partial \theta^{\prime }(t)}{\partial v}=\frac{d_{0}\sin\varphi_{0}(d_{0}-vt)(d_{0}+vt)}{{\left(d_{0}{}^{2}+v^{2}t^{2}-2d_{0}vt\cos\varphi_{0}\right)}^{2}}$$

**Figure 2:**
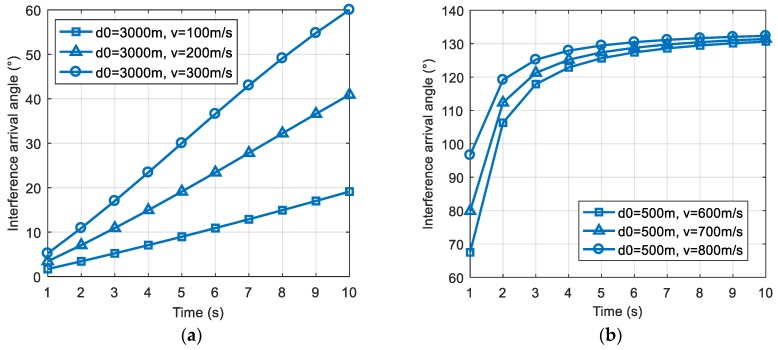
The change of the interference direction with the relative motion velocity v. (**a**) The change of the interference direction in the case where $vt<d_{0}$; (**b**) the change of the interference direction in the case where $vt>d_{0}$.

We analyze Equation (3) and find that, since $0^{{}^{\circ}}<\varphi_{0}<180^{{}^{\circ}}$, the positive and negative of $\frac{\partial \theta^{\prime }(t)}{\partial v}$ depends on $\left(d_{0}-vt\right)$. Therefore, when $vt<d_{0}$, $\frac{\partial \theta^{\prime }(t)}{\partial v}>0$, if v increases, the rate of change of the interference direction will also increase; when $vt>d_{0}$, $\frac{\partial \theta^{\prime }(t)}{\partial v}<0$, if v increases, the rate of change of the interference direction will decrease. We verified the above conclusions through the simulation of specific scenes. Figure 2a shows the case of $vt<d_{0}$, as v increased, the slope of the curve also increased. Figure 2b shows the case of $vt>d_{0}$, as v increased, the slope of the curve decreased.

Then, we take the partial derivative of $\theta^{\prime }(t)$ with respect to $d_{0}$ and get

(4) $$\frac{\partial \theta^{\prime }(t)}{\partial d_{0}}=\frac{v\sin\varphi_{0}(vt-d_{0})(vt+d_{0})}{{\left(d_{0}{}^{2}+v^{2}t^{2}-2d_{0}vt\cos\varphi_{0}\right)}^{2}}$$

**Figure 3:**
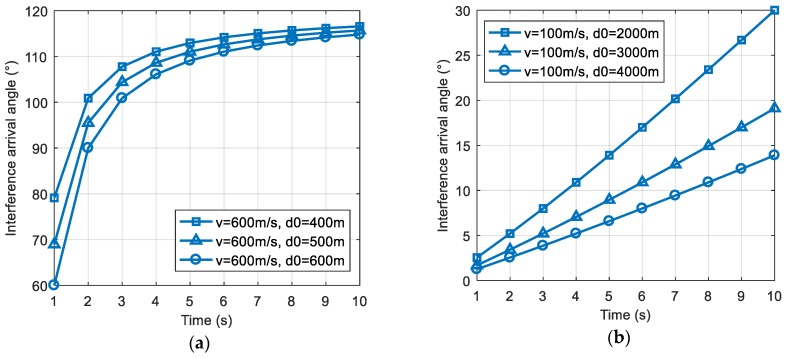
The change of the interference direction with the initial distance $d_{0}$. (**a**) The change of the interference direction in the case where $d_{0}<vt$; (**b**) the change of the interference direction in the case where $d_{0}>vt$.

Similarly, the positive and negative of Equation (4) depend on $\left(vt-d_{0}\right)$. When $d_{0}<vt$, $\frac{\partial \theta^{\prime }(t)}{\partial v}>0$, if $d_{0}$ increases, the rate of change of the interference direction will increase; when $d_{0}>vt$, $\frac{\partial \theta^{\prime }(t)}{\partial v}<0$, if $d_{0}$ increases, the rate of change of the interference direction will decrease. Figure 3a,b shows the scene simulation results for $d_{0}<vt$ and $d_{0}>vt$, respectively. We compared the slopes of the curves and the simulation results were consistent with the above analysis.

Finally, we take the partial derivative of $\theta^{\prime }(t)$ with respect to $\varphi_{0}$ and get

(5) $$\frac{\partial \theta^{\prime }(t)}{\partial \varphi_{0}}=\frac{d_{0}v\left[(d_{0}{}^{2}+v^{2}t^{2})\cos\varphi_{0}-2d_{0}vt\right]}{{\left(d_{0}{}^{2}+v^{2}t^{2}-2d_{0}vt\cos\varphi_{0}\right)}^{2}}$$

The positive and negative of Equation (5) depends on $(d_{0}{}^{2}+v^{2}t^{2})\cos\varphi_{0}-2d_{0}vt$, and we define

(6) $$\Psi =(d_{0}{}^{2}+v^{2}t^{2})\cos\varphi_{0}-2d_{0}vt$$

and take the derivative of Ψ with respect to $\varphi_{0}$ and get

(7) $$\Psi^{\prime }=-(d_{0}{}^{2}+v^{2}t^{2})\sin\varphi_{0}$$

In the interval of $0^{{}^{\circ}}<\varphi_{0}<180^{{}^{\circ}}$, $\Psi^{\prime }<0$, so Ψ monotonically decreases as $\varphi_{0}$ increases. Therefore, we can solve the value of $\varphi_{0}$ corresponding to Ψ=0 to determine the positive and negative boundary of $\frac{\partial \theta^{\prime }(t)}{\partial \varphi_{0}}$. When Ψ=0 in Equation (6), we can obtain

(8) $$\varphi_{0}=\arccos(\frac{2d_{0}vt}{d_{0}{}^{2}+v^{2}t^{2}})$$

**Figure 4:**
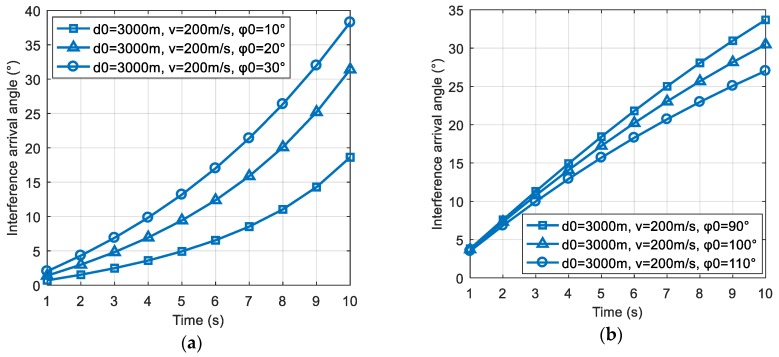
The change of the interference direction with the relative motion direction $\varphi_{0}$. (**a**) The change of the interference direction in the case where $\varphi_{0}<\arccos(\frac{2d_{0}vt}{d_{0}{}^{2}+v^{2}t^{2}})$; (**b**) the change of the interference direction in the case where $\varphi_{0}>\arccos(\frac{2d_{0}vt}{d_{0}{}^{2}+v^{2}t^{2}})$.

Therefore, when $\varphi_{0}<\arccos(\frac{2d_{0}vt}{d_{0}{}^{2}+v^{2}t^{2}})$, $\frac{\partial \theta^{\prime }(t)}{\partial v}>0$, if $\varphi_{0}$ increases, the rate of change of the interference direction will increase; and when $\varphi_{0}>\arccos(\frac{2d_{0}vt}{d_{0}{}^{2}+v^{2}t^{2}})$, $\frac{\partial \theta \prime (t)}{\partial v}<0$, if $\varphi_{0}$ increases, the rate of change of the interference direction will decrease. The scene simulation results are shown in Figure 4a,b.

Through the above analysis, we obtained the relationship between the rate of change of the interference direction and the speed of the relative motion, the direction of the relative motion, and the initial distance between the interference and receiver. Next, we simulated the corresponding scene to verify the phenomenon that the nulling of the spatial filtering becomes wider and shallower under dynamic scenes. First, we needed to introduce the basic principle of conventional spatial filtering.

**Figure 5:**
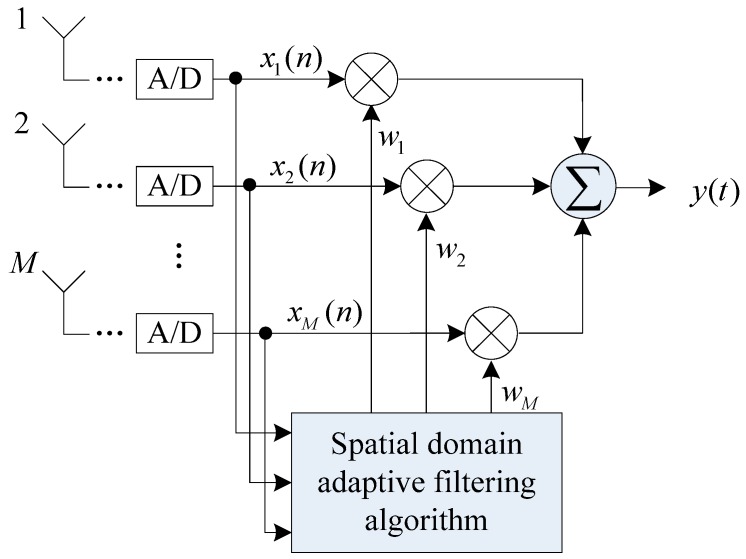
Schematic diagram of the spatial filtering structure based on the antenna array.

As shown in Figure 5, an antenna array was composed of M array elements. The signal received at the unit sampling time can be expressed as

(9) $$x={\left[x_{1}(n),x_{2}(n),\ldots ,x_{M}(n)\right]}^{H}$$

Assuming that the anti-jamming weight vector is calculated every L sampling moments, the corresponding sampling signal is

(10) $$X=[x_{1},x_{2},\ldots ,x_{L}]$$

and the weight vector corresponding to M array elements can be expressed as

(11) $$w={\left[w_{1},w_{2},\ldots ,w_{M}\right]}^{H}$$

We require that the power of the signal be minimal and not equal to 0 [13], i.e.,

(12) $$\begin{array}{l} \underset{w}{\min}\;\;\;w^{H}R_{x}w\\ s.t.\;\;\;w^{H}s=1 \end{array}$$

where $R_{x}=E[XX^{H}]$, s is a constraint vector. From Equation (12), we construct the Lagrangian function [14,15] and get

(13) $$w=\frac{R_{x}{}^{-1}s}{s^{H}R_{x}{}^{-1}s}$$

It should be noted that regarding the autocorrelation matrix $R_{x}$, in practice, we use the sample autocorrelation matrix and denote it with ${\hat{R}}_{x}$, and the anti-jamming weight vector w is then statistically optimal [16]. Therefore

(14) $$w=\frac{{\hat{R}}_{x}{}^{-1}s}{s^{H}{\hat{R}}_{x}{}^{-1}s}$$

The final output of the spatial filtering is

(15) $$y=w^{H}X$$

## 3. Analysis of Spatial Filtering Performance in Static and Dynamic Scenes

Based on the above conventional spatial filtering algorithm, we respectively simulated the process of spatial filtering to suppress interference under static and dynamic conditions. The simulation parameter settings are shown in Table 1.

### 3.1. Static Scene

**Figure 6:**
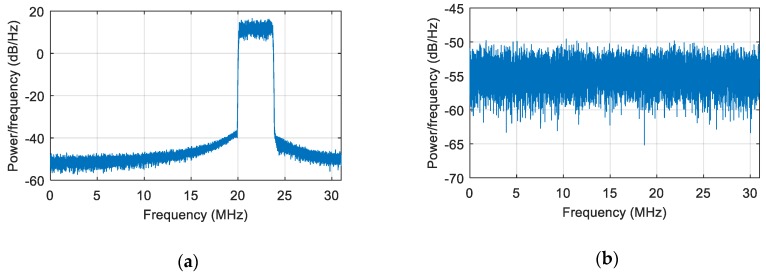
The power spectral density of the signal. (**a**) The power spectral density of the signal before interference suppression; (**b**) the power spectral density of the signal after interference suppression.

**Figure 7:**
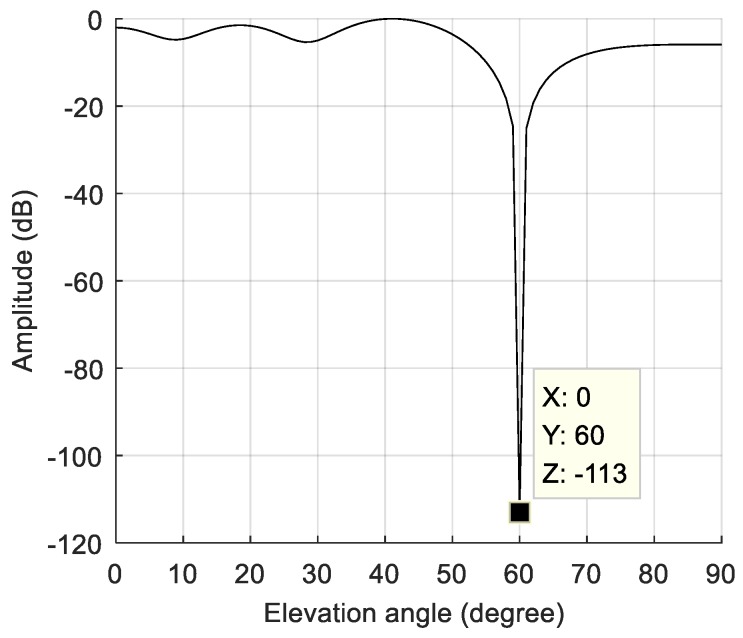
The null of the spatial filtering in the static scene.

**Figure 8:**
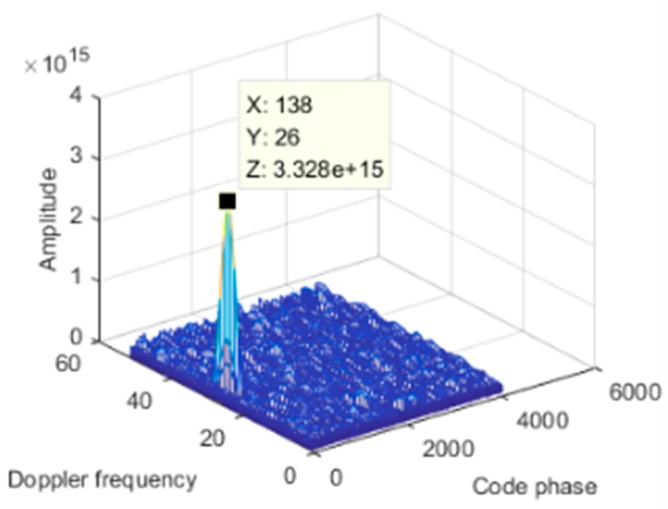
Acquisition result.

We first simulated a static scene, i.e., the interference and the receiver carrier were relatively stationary. We added a Gaussian interference with a jamming-to-signal ratio (JSR) of 80 dB. The power spectral density of the original signal is shown in Figure 6a. The power spectral density after spatial filtering is shown in Figure 6b. As seen from the figure, the interference was effectively suppressed, and the corresponding null is shown in Figure 7. At a pitch angle of 60°, a narrow null was formed, and the normalized null depth was –113 dB. As shown in Figure 8, there was an obvious correlation peak, and the signal was captured normally after anti-jamming.

### 3.2. Dynamic Scene

We set two dynamic scenes according to the dynamic model introduced above, a lower dynamic and a higher dynamic, generated corresponding satellite and interference signals, and then suppressed interference by spatial filtering.

#### 3.2.1. Lower Dynamic

**Figure 9:**
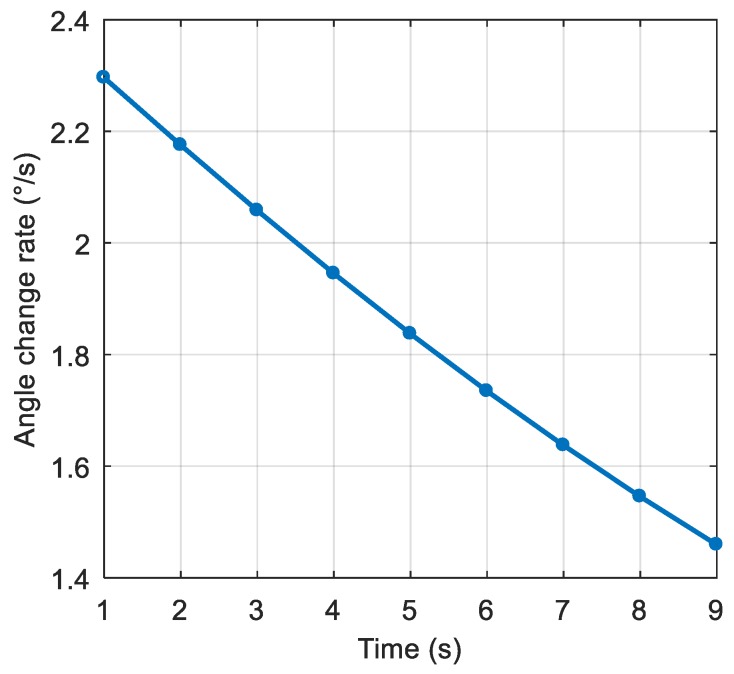
The rate of change of the interference arrival angle.

**Figure 10:**
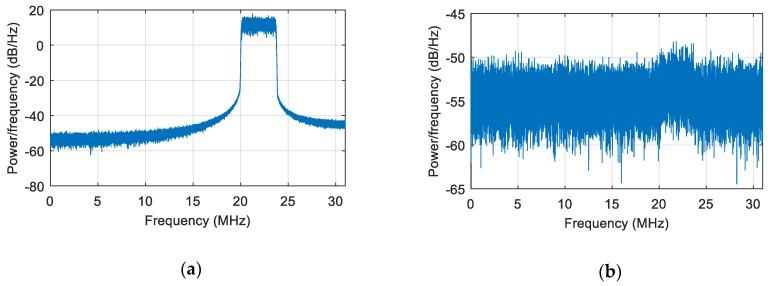
The power spectral density of the signal in the lower dynamic scene. (**a**) The power spectral density of the signal before interference suppression; (**b**) the power spectral density of the signal after interference suppression.

**Figure 11:**
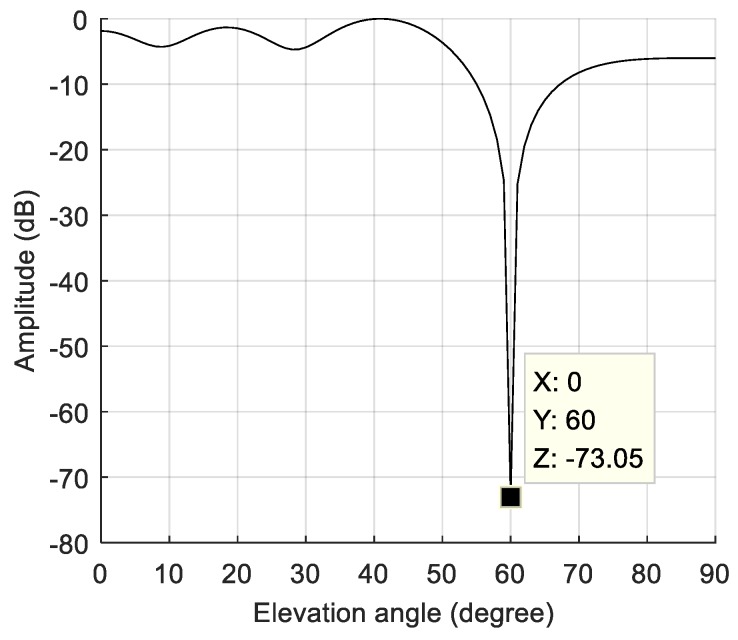
The null of the spatial filtering in the lower dynamic scene.

**Figure 12:**
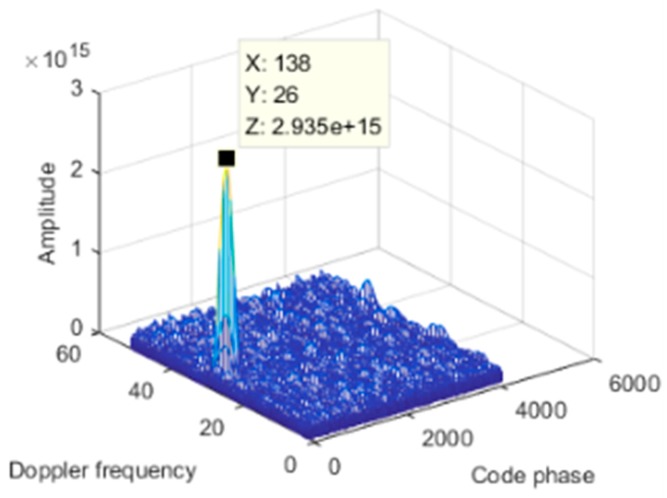
Acquisition result.

The dynamic model parameters are shown in Table 2. The corresponding rate of change of the interference arrival angle is shown in Figure 9. The anti-jamming weight vector was updated every millisecond, and in this scenario, the maximum rate of change of the interference arrival angle was approximately 0.0023 °/ms. The power spectral density of the signal after spatial filtering is shown in Figure 10b. It can be seen that the interference was basically filtered out, but the interference suppression was not very thorough compared with the static scene. Figure 11 shows the null corresponding to the spatial filtering. The variation of the null width was not obvious compared to the static scene, but the depth of the null was significantly reduced. The normalized depth was −73.05 dB, 39.5 dB lower than that of the static scene. The above results show that the lower dynamic also hadan impact on the spatial filtering. The shallower depth of the null affected the interference suppression, but the signal was still captured normally. The acquisition result is shown in Figure 12, and it can be seen that the correlation peak amplitude was slightly reduced relative to the static scene.

#### 3.2.2. Higher Dynamic

**Figure 13:**
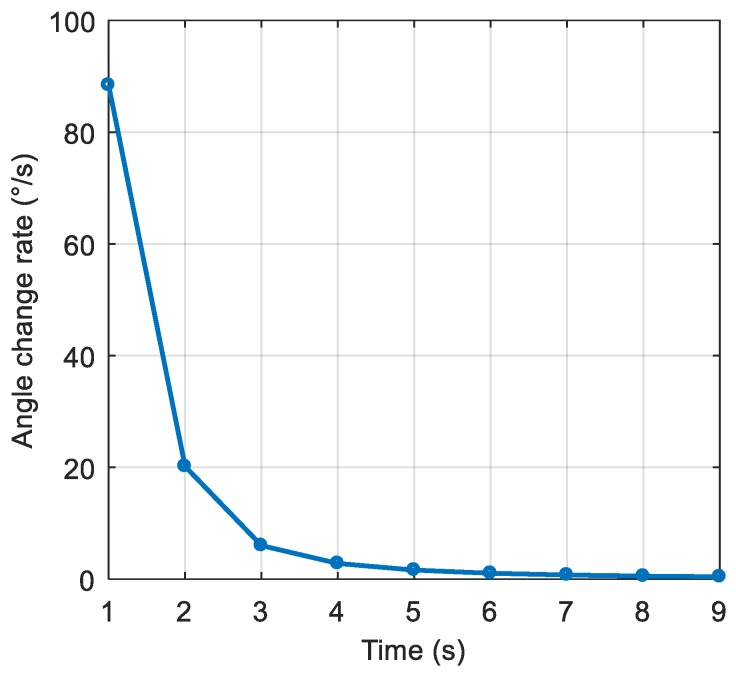
The rate of change of the interference arrival angle.

**Figure 14:**
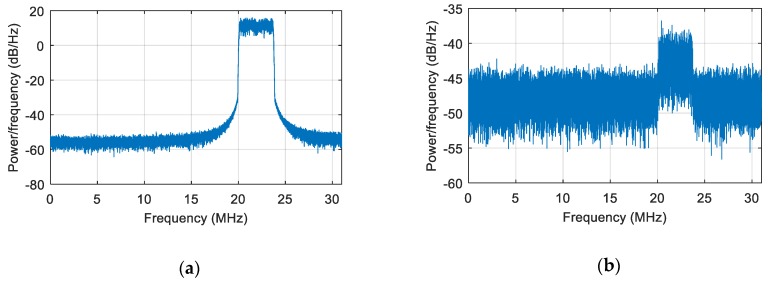
The power spectral density of the signal in the higher dynamic scene. (**a**) The power spectral density of the signal before interference suppression; (**b**) the power spectral density of the signal after interference suppression.

**Figure 15:**
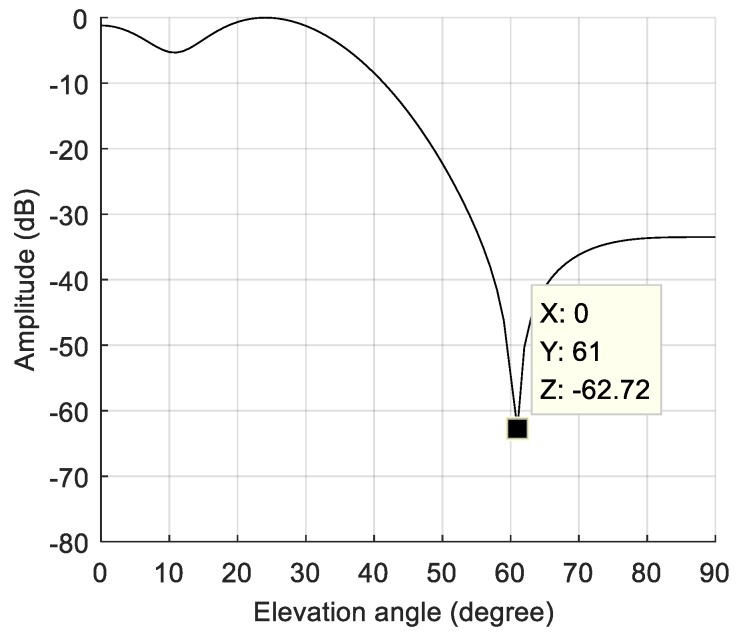
The null of the spatial filtering in the higher dynamic scene.

**Figure 16:**
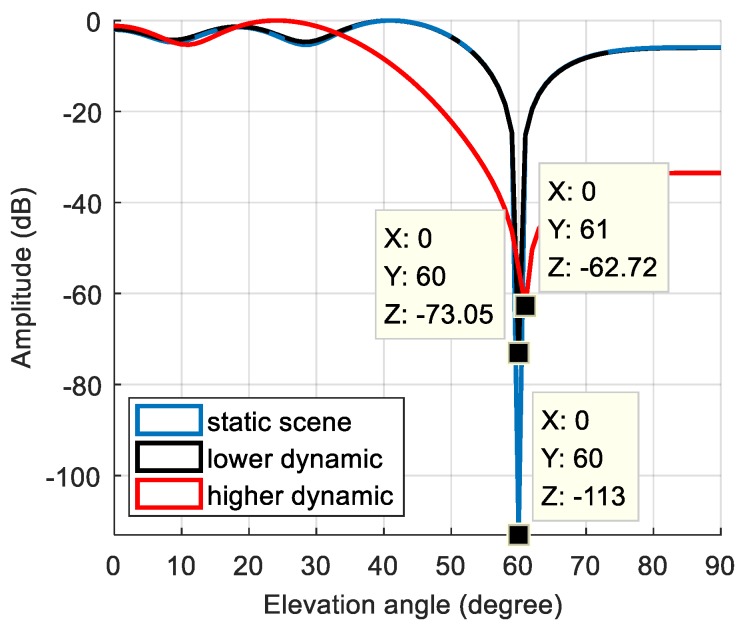
The null depth corresponding to the static scene, the lower dynamic scene and the higher dynamic scene.

**Figure 17:**
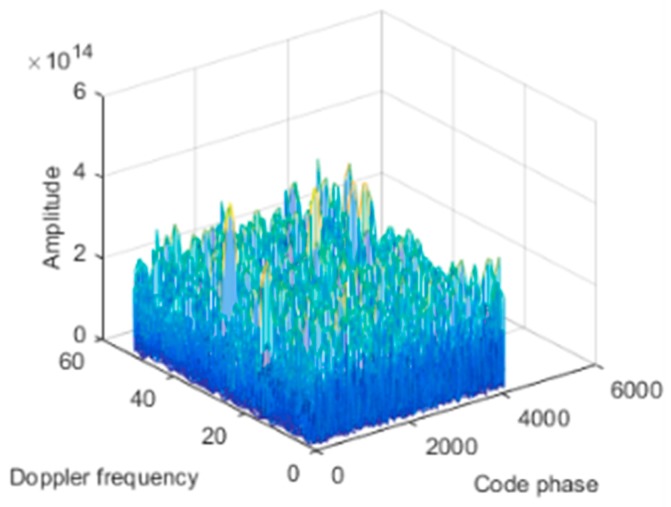
Acquisition result.

The dynamic model parameters are shown in Table 3. The corresponding rate of change of the interference arrival angle is shown in Figure 13. In this scenario, the maximum rate of change of the interference arrival angle reached 0.09 °/ms. Figure 14a shows the power spectral density of the signal before anti-jamming and Figure 14b shows the power spectral density of the signal after anti-jamming. The effect of interference suppression was significantly worse than that of static and lower dynamic scenes, and there was significant residual interference. The null corresponding to the spatial filtering is shown in Figure 15. The width of the null was obviously widened, and the normalized depth was only −62.72 dB. As shown in Figure 16, we compared the null depth corresponding to the static scene, the lower dynamic scene, and the higher dynamic scene. It can be seen that the depth of the null in the higher dynamic was further reduced compared to the lower dynamic scene. Therefore, after the rate of change of the interference direction increased, the spatial filtering performance further deteriorated, and the null became wider and shallower, which means that the interference was not completely suppressed; further, the wider null may have also affected the satellite signal. Figure 17 shows the signal acquisition result after interference suppression, and there was no obvious correlation peak in the figure, so the signal was not captured normally.

## 4. Interference-Nulling Control Algorithm

For the dynamic scene, a shallow nulling will result in poor spatial filtering performance. Therefore we propose a dual projection method to deepen the nulling and improve the performance of the spatial filtering in dynamic scenes.

**Figure 18:**
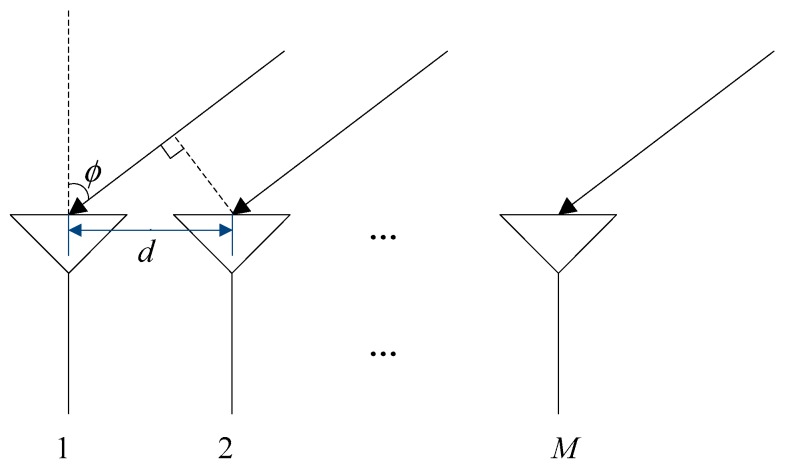
Antenna array schematic.

First, we solve the anti-interference vector normally according to the conventional spatial filtering algorithm, and find the spatial spectral function of the corresponding anti-jamming vector,

(16) $$F(\phi )=w^{H}a(\phi )$$

where ϕ represents the angle of arrival, and a(ϕ) represents the steering vector of the signal [17,18].

(17) $$a(\phi )={\left[1,e^{-\frac{j2\pi d\sin\phi }{\lambda }},\ldots ,e^{-\frac{j2\pi (M-1)d\sin\phi }{\lambda }}\right]}^{T}$$

as shown in Figure 18, d represents the element spacing, M represents the number of array element, λ represents the signal wavelength. Then, we traverse ϕ from 0°–90°, calculate the value of the corresponding F(ϕ), and then find the minimum value of F(ϕ).The corresponding ϕ is the approximate position of the interference nulling. However, the nulling was wider and shallower, so we deepened the nulling based on the double projection method.

After ϕ is obtained, from Equation (17), we can obtain the steering vector of the interference signal,

(18) $$A=[a(\phi_{1}),a(\phi_{2}),\ldots ,a(\phi_{N})]$$

where N is the number for the interference. Since the generated nulling was not deep enough, according to the spatial filtering algorithm, we hoped to increase the proportion of interference in the signal. Therefore, we projected the signal into the interference subspace and deepened the sampling of the interference signal [19,20]. The projection matrix is

(19) $$P_{a}=A{\left(A^{H}A\right)}^{-1}A^{H}$$

Combined with Equation (10), we can obtain the deepened sampled signal,

(20) $$X_{D}=X+uP_{a}X$$

where u is the nulling control coefficient. Then, the autocorrelation matrix of the deepened sampled signal can be obtained, i.e.,

(21) $$R_{x_{D}}=E[X_{D}X_{D}^{H}]$$

and the corresponding anti-jamming weight vector is

(22) $$w_{D}=\frac{R_{x_{D}}^{-1}s}{s^{H}R_{x_{D}}^{-1}s}$$

Then, we consider the second projection to further deepen the depth of the nulling. Because the interference signal subspace is orthogonal to the noise subspace, we can project the anti-jamming weight vector $w_{D}$ to the noise subspace to further improve the orthogonality between $w_{D}$ and the interference signal subspace. To do this, we need to obtain the corresponding projection matrix. First, after the eigen decomposition of $R_{x_{D}}$, we can get

(23) $$R_{x_{D}}=\sum_{i=1}^{M}\lambda_{i}v_{i}v_{i}^{H}=U_{j}\Lambda_{j}U_{j}^{H}+U_{n}\Lambda_{n}U_{n}^{H}$$

where $\lambda_{i}$ represents eigenvalue, and M represents the number of array element.

(24) $$\Lambda_{j}=\left[\begin{array}{cccc} \lambda_{1} & & & \\ & \lambda_{2} & & \\ & & \ddots & \\ & & & \lambda_{K} \end{array}\right]$$

is a diagonal matrix of K large eigenvalues. $U_{j}=[v_{1},v_{2},\cdots ,v_{K}]$ represents the interference signal subspace formed by the eigenvectors corresponding to the K large eigenvalues.

(25) $$\Lambda_{n}=\left[\begin{array}{cccc} \lambda_{K+1} & & & \\ & \lambda_{K+2} & & \\ & & \ddots & \\ & & & \lambda_{M} \end{array}\right]$$

is a diagonal matrix of M−K smaller eigenvalues (The eigenvalues are arranged in descending order. If the adjacent eigenvalues exceed one order of magnitude, the corresponding K can be used as the boundary value of eigenvalues). $U_{n}=[v_{K+1},v_{K+2},\cdots ,v_{M}]$ represents the noise subspace formed by the eigenvectors corresponding to the M−K eigenvalues [21,22]. According to the noise subspace $U_{n}$, the corresponding projection matrix can be obtained,

(26) $$P_{n}=U_{n}{\left(U_{n}{}^{H}U_{n}\right)}^{-1}U_{n}{}^{H}$$

Then, the anti-jamming weight vector $w_{D}$ is projected onto the noise subspace to obtain the anti-jamming weight vector after double projection,

(27) $$w_{DD}=P_{n}w_{D}$$

The final output is

(28) $$y=w_{DD}^{H}X$$

**Figure 19:**
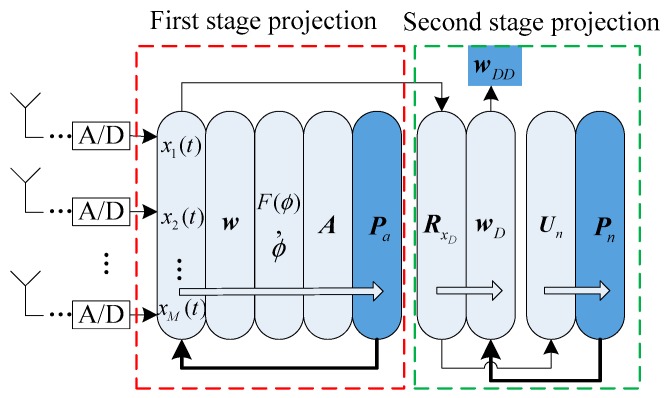
The schematic diagram of the signal processing flow of the double projection algorithm.

The signal processing flow of the double projection algorithm was as follows, as shown in Figure 19:

1. Solve the anti-jamming weight vector w according to spatial filtering algorithm;
2. Calculate the spatial spectral function F(ϕ) about the anti-jamming vector and determine the direction angle ϕ corresponding to the null, and the steering vector A of the interference signal can be obtained;
3. Calculate the projection matrix $P_{a}$, project the sampled signal into the noise subspace and calculate the autocorrelation matrix $R_{x_{D}}$, then calculate the anti-jamming vector $w_{D}$;
4. Perform eigen decomposition on $R_{x_{D}}$, construct a noise subspace $U_{n}$, and calculate the corresponding projection matrix $P_{n}$;
5. Project the anti-jamming weight vector $w_{D}$ to the noise subspace and obtain $w_{DD}$.

## 5. Simulation and Test

We simulated the dynamic scene and deepened the nulling based on the dual projection method to verify the improvement effect of the proposed method on the spatial filtering performance.

**Figure 20:**
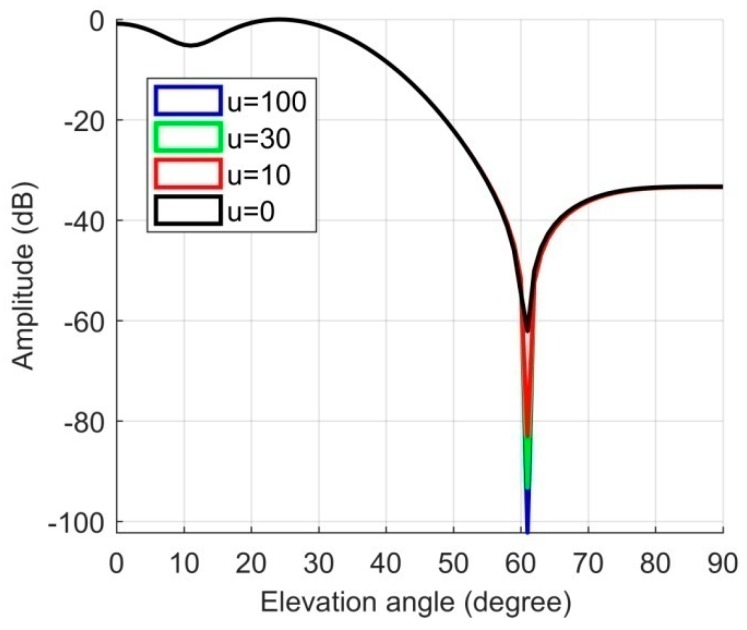
The corresponding nulling for different nulling control coefficients.

**Figure 21:**
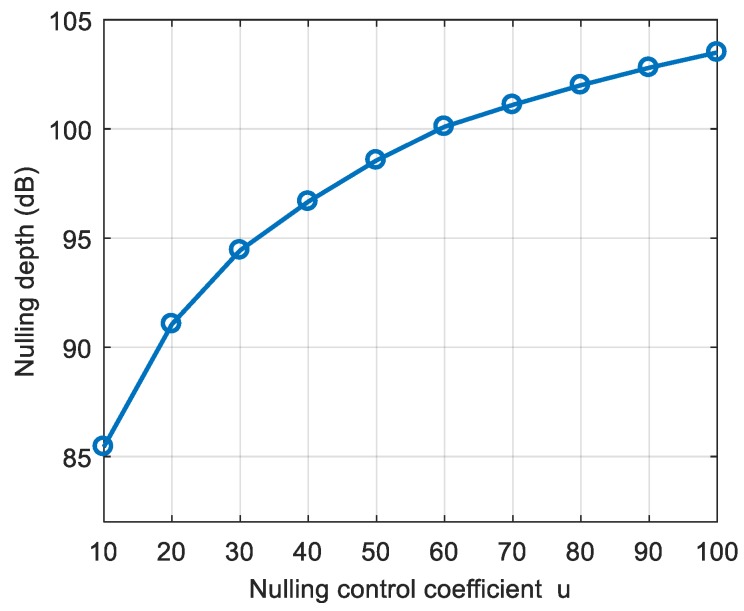
The corresponding nulling depths for different nulling control coefficients.

The simulated scene parameters were the same as those in the higher dynamics in the third section, and the maximum rate of change of the interference arrival angle was 0.09 °/ms. First, through the first projection, the sampling of the interference signal was deepened, and then the anti-jamming weight vector was obtained. Figure 20 shows the corresponding nulling obtained by different nulling control coefficient u, where u=0 was the nulling depth corresponding to the original spatial filtering algorithm, which was −62.72 dB. The depth of the nulling after the first projection was significantly deepened. When u=10, the nulling depth exceeded −80 dB, and when u=100, the nulling depth exceeded −100 dB. Therefore, the depth of nulling could be increased by at least 20–30 dB after the first projection. It can be seen from Figure 21 that as u increased, the depth of the nulling gradually deepened, but the magnitude of the increase gradually decreased. Therefore, u could be as large as possible, but the deepening effect on the nulling was gradually stabilized. In this paper, we generally set u to 50–100.

**Figure 22:**
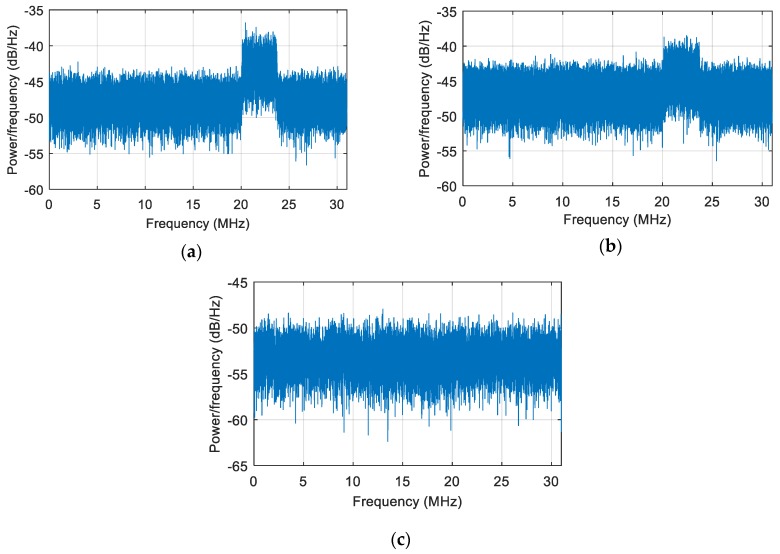
The power spectral density of the signal. (**a**) The signal power spectral density obtained by the original spatial filtering; (**b**) the signal power spectral density obtained by the spatial filtering after the first projection; (**c**) the signal power spectral density obtained by spatial filtering after dual projection.

We took u=50 and solved the anti-jamming weight vector for spatial filtering. The power spectral density after anti-jamming is shown in Figure 22b, and the power spectral density obtained by the original spatial filtering is shown in Figure 22a. By comparison, it can be seen that the interference was better suppressed, but the suppression was still not thorough enough.

**Figure 23:**
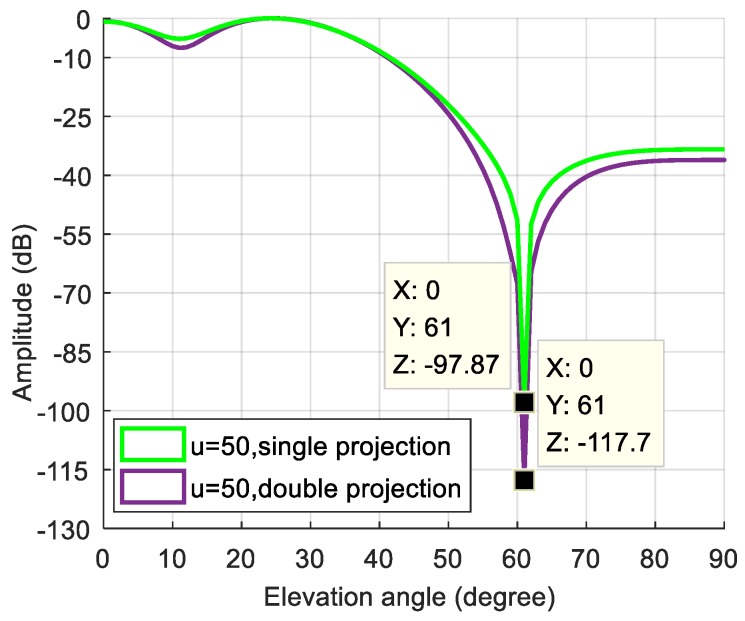
The depth of the nulling corresponding to single projection and double projection.

**Figure 24:**
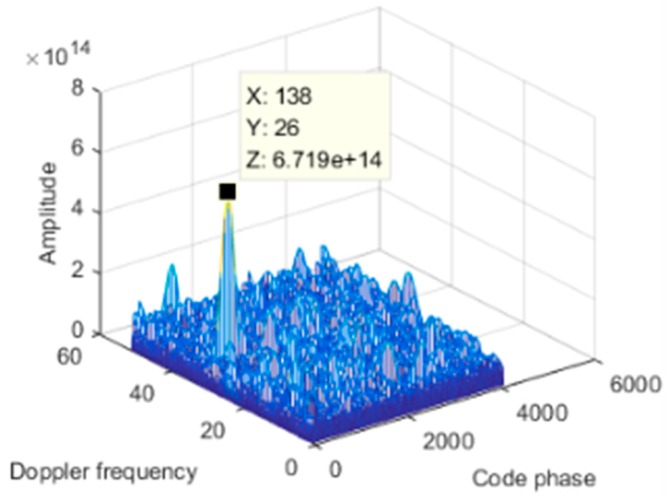
Acquisition result.

To further deepen the nulling, we used dual projection, i.e., based on the first projection, and the anti-jamming weight vector was projected to the noise subspace. As shown in Figure 23, the depth of the null after double projection was further deepened from −97.87 dB to −117.7 dB. Figure 22c shows the corresponding signal power spectral density after spatial filtering based on dual projection. Compared with Figure 22b, the interference was suppressed completely. The acquisition result is shown in Figure 24, and there is an obvious correlation peak in the figure, which indicates that the signal can be captured normally, it can be seen that the correlation peak amplitude is also slightly reduced relative to the static scene. Overall, the double projection method was used to deepen the nulling, which effectively ensured and improved the anti-jamming performance of the spatial filtering in dynamic scenes.

## 6. Conclusions

In this paper, a dynamic model between the interference and the receiver was established, and the defect that the null depth of the spatial filtering was shallow in the dynamic scene was verified. A null control algorithm based on double projection was proposed. The simulation results showed that the proposed algorithm had stable control ability for nulls, and the depth of the null could be deepened by at least 30 to 50 dB, thus ensuring and improving the anti-interference performance of the spatial filtering in dynamic scenes.

## Figures and Tables

**Table 1:** Simulation parameter settings.

Category	Parameter	Value
Satellite signal parameters	Sampling frequency	62 MHz
	RF frequency	1561.098 MHz
	Intermediate frequency	40.098 MHz
	Code rate	2.046 MHz
	Signal bandwidth	4.092 MHz
	Carrier-to-noise ratio	45 dBHz
	Signal direction (angle of pitch)	40°
Interference parameters	Type of interference	Gaussian interference
	Interference bandwidth	4.092 MHz
	Jamming-to-signal ratio (JSR)	80 dB
	Initial direction of interference (angle of pitch)	60°
Array parameters	Array type	Linetype
	Number of array elements	7
	Element spacing	Half-wavelength
Anti-jamming algorithm parameter	Weight vector update time	1 ms

**Table 2:** Dynamic parameter setting.

Symbol	Parameter	Value
v	Relative speed	100 m/s
$\varphi_{0}$	The angle between the relative motion direction and the radial connection	120°
$d_{0}$	Initial distance	2000 m
$\theta^{\prime }(t)$	Maximum rate of change of the interference arrival angle	0.0023 °/ms

**Table 3:** Dynamic parameter setting.

Symbol	Parameter	Value
v	Relative speed	700 m/s
$\varphi_{0}$	The angle between the relative motion direction and the radial connection	20°
$d_{0}$	Initial distance	1000 m
$\theta^{\prime }(t)$	Maximum rate of change of the interference arrival angle	0.09 °/ms
